# The effect of abatacept on T-cell activation is not long-lived *in vivo*

**DOI:** 10.1093/discim/kyad029

**Published:** 2024-01-04

**Authors:** Larissa C da Rosa, Hannah E Scales, Robert A Benson, James M Brewer, Iain B McInnes, Paul Garside

**Affiliations:** School of Infection & Immunity, College of Medical, Veterinary and Life Sciences, University of Glasgow, Glasgow G12 8TA, UK; School of Infection & Immunity, College of Medical, Veterinary and Life Sciences, University of Glasgow, Glasgow G12 8TA, UK; School of Infection & Immunity, College of Medical, Veterinary and Life Sciences, University of Glasgow, Glasgow G12 8TA, UK; School of Infection & Immunity, College of Medical, Veterinary and Life Sciences, University of Glasgow, Glasgow G12 8TA, UK; School of Infection & Immunity, College of Medical, Veterinary and Life Sciences, University of Glasgow, Glasgow G12 8TA, UK; School of Infection & Immunity, College of Medical, Veterinary and Life Sciences, University of Glasgow, Glasgow G12 8TA, UK

**Keywords:** Abatacept, CTLA-4Ig, cell interaction, costimulatory molecules

## Abstract

Abatacept, a co-stimulatory blocker comprising the extracellular portion of human CTLA-4 linked to the Fc region of IgG1, is approved for the treatment of rheumatoid arthritis. By impairing the interaction between CD28 on T cells and CD80/CD86 on APCs, its mechanisms of action include the suppression of follicular T helper cells (preventing the breach of self-tolerance in B cells), inhibition of cell cycle progression holding T cells in a state described as ‘induced naïve’ and reduction in DC conditioning. However, less is known about how long these inhibitory effects might last, which is a critical question for therapeutic use in patients. Herein, employing a murine model of OVA-induced DTH, we demonstrate that the effect of abatacept is short-lived *in vivo* and that the inhibitory effects diminish markedly when treatment is ceased.

## Graphical Abstract

**Graphical Abstract:**
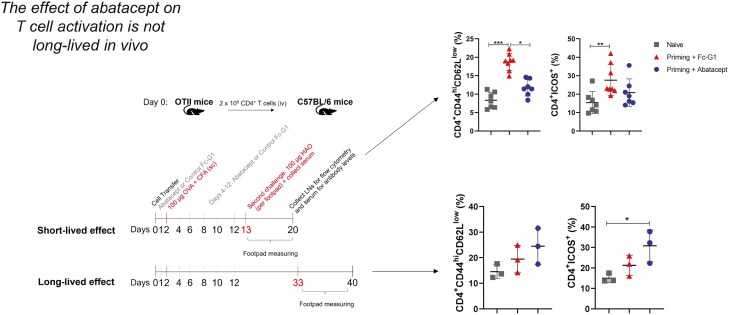

## Introduction

At the onset of adaptive immune responses lays the interaction between naïve CD4 T cells and dendritic cells (DCs), resulting in T-cell priming. Along with the first signal exchanged by these two cells, i.e. the antigen presentation from the DC major histocompatibility complex (MHC) II to the T-cell receptor (TCR), a second signal needs to be provided by costimulatory molecules to enhance TCR response [1]. The most important costimulatory interaction for the activation of these naïve CD4 T cells is the binding of CD28 with CD80/86 on DCs. When both signals are sufficient, T cells express activation markers, secrete cytokines, and their cell cycle progresses into proliferation and differentiation [2, 3]. On the other hand, cytotoxic T-lymphocyte-associated protein 4 (CTLA-4) is structurally similar to CD28 but binds to CD80 and CD86 with higher affinity and avidity, promoting anergy, and suppressing T-cell activation. It is also essential for Foxp3 regulatory T-cell function [4, 5].

Abatacept, a fusion protein comprising a modified Fc portion of human IgG1 and the extracellular domain of the CTLA-4 molecule (reviewed in [6]), binds to CD80/CD86 on APCs, impairing CD28 binding and preventing optimal T-cell activation. Previous studies demonstrated that its mode of action includes the downregulation of activation markers and reduction of proinflammatory cytokine secretion. Furthermore, suppression of the acquisition of a T follicular helper cell (T_fh_) phenotype prevents T-cell migration into the B-cell area in the lymph nodes, inhibiting B-cell response and antibody production [7]. Investigating the transcriptional profile of treated T cells, studies demonstrated that abatacept stops their cell cycle between TCR engagement and priming, in a state termed T_induced naïve_ [8]. These T_induced naïve_ cells lead to reduced APC conditioning, altering subsequent T-cell activation [8].

Abatacept has already been approved for the treatment of rheumatoid arthritis (RA), juvenile idiopathic arthritis, and active psoriatic arthritis, while it is also being tested in clinical trials for acute graft-versus-host disease, type 1 diabetes, and multiple sclerosis, among other autoimmune diseases [9]. Therefore, it is a drug that has been consolidated as an option in various treatments, with promising outcomes and proved safety. However, some of the challenges with abatacept treatment are the reduction in the number of regulatory T cells in patients, as the drug does not have the ability to induce Tregs like the membrane-bound CTLA-4 [10], and how effective it can be on established diseases when the need for CD28 co-stimulation for T-cell activation is reduced. Scarsi *et al.* [11] demonstrated that lower numbers of circulating CD4^+^CD28^null^ cells at the onset of treatment could predict better efficacy, and Heinbokel *et al.* [12] showed that older mice, which have higher frequencies of CD28^null^ T cells, were more prone to organ rejection. Thus, critical questions regarding the duration of the effect of abatacept *in vivo* and the most appropriate timing for treatment remain unanswered. In this study, we investigated these parameters using a murine OVA-induced delayed-type hypersensitivity (DTH) model.

## Materials and methods

### Mice

Eight- to 10-week-old female C57BL/6 mice were purchased from Envigo (Bicester, UK). OT-II T-cell receptor (TCR) transgenic mice were bred in-house. This TCR is specific for the chicken ovalbumin peptide 323-339, presented in the context of I-A^b^ MHC II molecules. Mice were housed at the University of Glasgow and maintained under standard animal house conditions. All procedures were conducted in accordance with UK Home Office regulations.

### Adoptive transfer

Spleen and peripheral lymph nodes were collected from OT-II transgenic mice and processed for single-cell suspensions. The percentage of CD4^+^CD45.1^+^Vα2^+^Vβ5^+^ cells was determined by flow cytometry, and 2 × 10^6^ naïve OT-II CD4 T cells were transferred intravenously (iv) into C57BL/6 mice on day 0, for all protocols.

### Reagents

Abatacept (CTLA-4Ig) was provided by Bristol-Myers Squibb. For the control group, recombinant human IgG1 Fc (Fc-G1; BioXCell – Pennsylvania, USA) was used. Both were injected at 10 mg/kg, intraperitoneally (ip). Following previous protocols [8], the first injection was administered one day prior to immunization, followed by three-to-five injections afterwards, with a 2-day interval.

### Immunizations

#### First immunizations

Two days after the adoptive transfer, mice were injected subcutaneously (sc) in the scruff of the neck with either 100 µg of ovalbumin (OVA, Sigma-Aldrich), emulsified (1:1) in Complete Freund’s Adjuvant (CFA, Sigma-Aldrich) or 100 µg of OVA and 10 µg of LPS (lipopolysaccharides from *Escherichia coli* O111:B4; Sigma-Aldrich) in phosphate-buffer saline (PBS).

#### Induction of DTH

The challenge with heat-aggregated OVA (HAO) into the footpads (100 µg HAO/footpad) was performed at different time points, depending on the protocol, but varying from 10 to 21 days post OVA/CFA immunizations [13]. Naïve mice (negative controls) were not injected.

#### Short-term immunization

**Figure 1:**
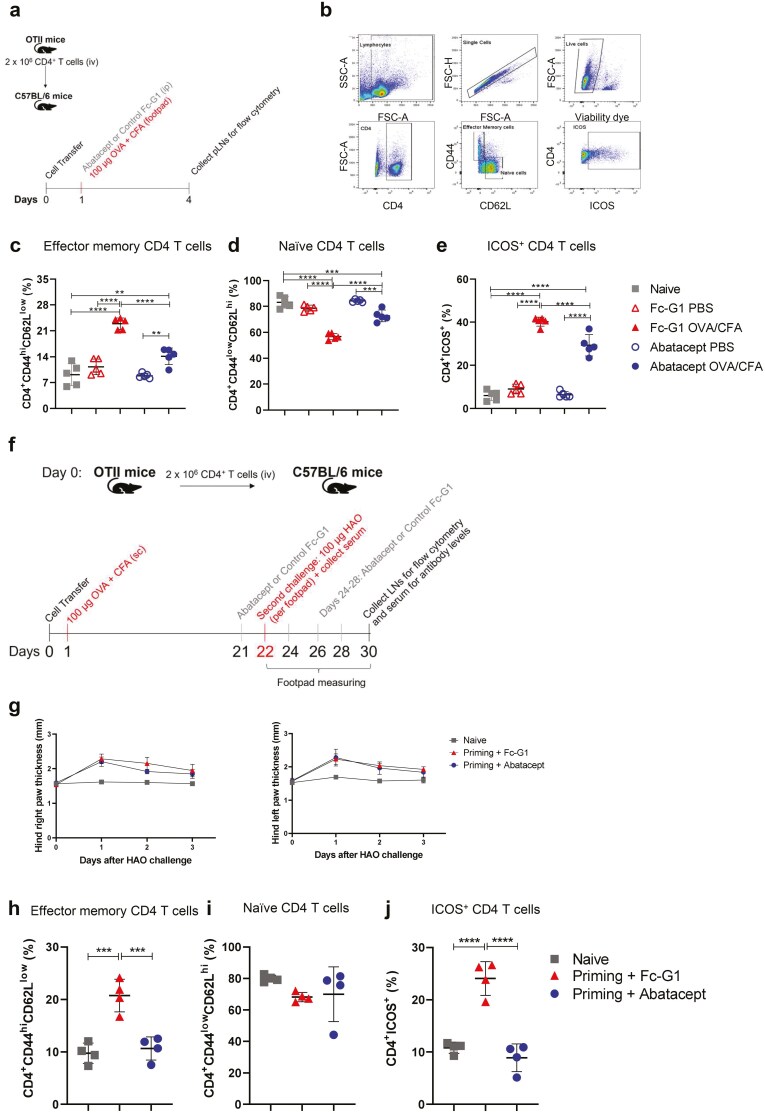
**A single dose of Abatacept at the time of priming reduces effector CD4** ^**+**^ **T cell proportion and the effect of administration of abatacept on established immune responses.** (**A**) OTII cells were transferred into female C57BL/6 mice. One day later, mice were injected with either abatacept or Fc-G1 (control antibody) and immediately immunized into the right footpads with OVA/CFA. The left foot was injected with PBS, as a negative control. Negative control mice (Naïve group) were not immunized. Three days after immunization, popliteal lymph nodes were collected for flow cytometry. (**B**) Gating strategy for flow cytometry data. Single, live CD4 T cells were distinguished as naïve (CD4^+^CD44^low^CD62L^hi^) or effector memory (CD4^+^CD44^hi^CD62L^low^) and gated for the expression of ICOS. (**C**) Percentage of effector memory CD4^+^ T cells. (**D**) Percentage of naïve CD4^+^ T cells. (**E**) Percentage of CD4^+^ T cells expressing ICOS. Grey squares represent naïve mice; empty red triangles represent draining lymph nodes from PBS-footpads in mice injected with Fc-G1, and filled red triangles represent draining lymph nodes from OVA/CFA-footpads injected with Fc-G1; empty blue circles represent draining lymph nodes from PBS-footpads in mice injected with abatacept and filled blue circles represent draining lymph nodes from OVA/CFA-footpads injected with abatacept. (**F**) To investigate the effect of abatacept on established immune responses, OTII cells were transferred into female C57BL/6 mice and, one day later, they were immunized subcutaneously with OVA/CFA. On day 21, mice received the first dose of either abatacept or Fc-G1. One day later, mice were challenged into the footpads with HAO. For the posterior 7 days, mice received abatacept or Fc-G1 every other day. Negative control mice (Naïve group) were not immunized or challenged. By day 7, popliteal lymph nodes were collected for flow cytometry and blood serum for the detection of anti-OVA IgG levels. (**G**) Right and left, respectively, hind paw thickness was measured for 3 days after HAO injection with a calliper. (**H**) Percentage of effector memory CD4^+^ T cells. (**I**) Percentage of naïve CD4^+^ T cells. (**J**) Percentage of CD4^+^ T cells expressing ICOS. Grey squares represent naïve mice; red triangles represent mice injected with Fc-G1, and blue circles represent mice injected with abatacept. Data were analysed by one-way ANOVA, followed by post hoc Tukey’s test, for multiple comparisons. *P < 0.05; **P < 0.01; ***P < 0.001; ****P < 0.0001 and *n* = 5, over 1 experiment.

For the short-term experiment (Fig. 1), 24 h after adoptive transfer, mice received one single dose of either abatacept or Fc-G1 (10 mg/kg) ip and were injected into the right footpads with 100 µg of ovalbumin emulsified in CFA (1:1). Left footpads were injected with PBS for negative control.

### Assessment of DTH

Assessment of the DTH progression was done for 4 days, including the day of HAO immunization, by measuring paw thickness with a calliper (Kroeplin GmbH; Schlüchtern, Germany), according to previous protocols [14].

### *In vitro* restimulation of CD4 T cells

Cell suspensions were prepared from axillary, brachial, and inguinal lymph nodes, and 5 × 10^5^ total cells were cultured (5% CO_2_, 37°C) with 1 µg/ml of OVA peptide (OVA_323–339_) for 48 h. After this period, cells were stained for flow cytometry.

### Flow cytometry

Draining lymph nodes were collected, and single-cell suspensions were prepared and divided into two FAC tubes, to be stained for two different panels (one with surface markers for T cells and one for APCs). Timings for lymph node collection are detailed in the figure legends and the Results section. Briefly, cells were first stained with the viability dye (eF506, eBioscience), blocked for non-specific FcR binding, and then incubated with a master mix of the fluorochrome-conjugated antibodies for 30 min, at 4°C. The markers on the T-cell panel were CD4 (FITC, Invitrogen), CD44 (PerCP Cy5.5, Invitrogen), CD62L (e450, Invitrogen), and ICOS (PeCy7, Biolegend). The APC panel comprised: CD19 (PerCP Cy5.5, Invitrogen), CD11c (e450, Invitrogen), MHC II (H-2^b^; BV786, Biolegend), CD80 (FITC, BD Biosciences), and CD86 (PeCy7, Biolegend). Data were acquired on either BD LSRFortessa or BD LSR II (BD Biosciences) and analysed using FlowJo 10 software (Tree Star).

### Anti-OVA enzyme-linked immunosorbent assay (ELISA)

Anti-OVA IgG1 and IgG2c serum levels were measured by ELISA, as previously described in [13].

### Data analysis

Power calculations were performed by GPower 3. 1 software (Universität Kiel, Germany) and took into consideration the minimum number of mice necessary to provide the statistical difference between the groups for the frequency of CD4^+^ICOS^+^ cells. Experiments throughout this study passed the Shapiro–Wilk test for normality.

Results are shown as individual data, with a black bar representing the mean value ± SD. Data were analysed by one- or two-way analysis of variance (ANOVA), followed by post hoc Tukey’s test, for multiple comparisons. The *P* value adopted to establish a significant difference was *P* < 0.05. Statistical analysis was performed using Prism 8.3 (GraphPad).

## Results

### A single dose of abatacept at the time of priming reduces effector CD4^+^ T-cell proportion

We previously showed in DO11.10 TcR transgenic mice that administration of abatacept during priming reduced proliferation and the percentage of effector CD4^+^ T cells, as well as the levels of anti-OVA and anti-collagen antibodies, in a breach of the self-tolerance model of inflammatory arthritis [7, 8]. We first confirmed these findings. CD4^+^ T cells from OTII transgenic mice were transferred into C57BL/6 mice. On the next day, they received a single dose of either abatacept or control Fc-G1, ip, and immediately were immunized with OVA/CFA into their right footpad (control footpads were injected with PBS). Naïve mice, which received neither the OVA/CFA injection nor treatment, were included as negative controls. Three days later, popliteal lymph nodes (pLN) were collected for flow cytometry analysis (Fig. 1a).

To investigate T-cell activation status, single live CD4^+^ T cells were distinguished as effector memory (CD44^hi^CD62L^low^) or naïve (CD44^low^CD62L^hi^) cells and assessed on their percentage of ICOS (inducible T cell co-stimulator, a member of the CD28 superfamily) expression (Fig. 1b). There was a significant increase in the percentage of effector memory cells in the OVA/CFA pLN of mice treated with Fc-G1, compared with the PBS control pLN and naïve mice. There was also a significantly lower percentage of effector memory CD4^+^ T cells in the OVA/CFA pLN of mice treated with abatacept compared with those treated with Fc-G1, although higher than naïve mice (Fig. 1c). Conversely, there was a significant decrease in the percentage of naïve cells in the Fc-G1-OVA/CFA pLN, compared with PBS control-pLN, abatacept-treated mice and naïve mice (Fig. 1d). The percentage of CD4^+^ T cells expressing ICOS significantly increased following OVA/CFA immunization in both Fc-G1 and abatacept-treated groups; however, this was significantly reduced in mice treated with abatacept compared with the Fc-G1 control-treated mice (Fig. 1e).

Analysing the OVA-specific CD4^+^ T cells (Supplementary Fig. S1a) showed accumulation of CD4^+^CD45.1^+^ cells in immunized footpads of mice treated with abatacept (Supplementary Fig. S1b). We observed the same reductions in effector memory, naïve and ICOS^+^ cells (Supplementary Fig. S1c–e, respectively), as noted in total CD4^+^ T cells.

### Some aspects of established immune responses are affected by administration of abatacept

As well as studying the effect of one single dose of abatacept during priming, we wanted to investigate longer-term administration. Thus, OTII cells were transferred into C57BL/6 mice, and 24 hours later, they were immunized with OVA/CFA. After 21 days, mice received the first dose of abatacept or Fc-G1 and, on the next day, challenged with HAO in both footpads. During the following 7 days, mice were treated with abatacept or Fc-G1 every 2 days. Footpad thickness was measured during the first 3 days after the HAO challenge and pLNs were collected for flow cytometry on the eighth-day post challenge (Fig. 1f). Naïve, unimmunized, and untreated mice were used as negative controls.

No differences in paw thickness were observed between Fc-G1- and abatacept-treated groups (Fig. 1g). We observed that mice treated with abatacept had a significant reduction in the percentage of effector memory CD4^+^ T cells, compared with mice treated with Fc-G1 (Fig. 1h), while the percentage of naïve CD4^+^ T cells were similar between the groups (Fig. 1i). Abatacept treatment also reduced the frequency of CD4^+^ T cells expressing ICOS, compared with mice that were primed, but received Fc-G1 (Fig. 1j). For this protocol, the percentage of CD4^+^CD45.1^+^ cells retained in the pLNs was extremely small in all groups (Supplementary Fig. S1f) and, for that reason, their phenotype was not analysed.

**Figure 2:**
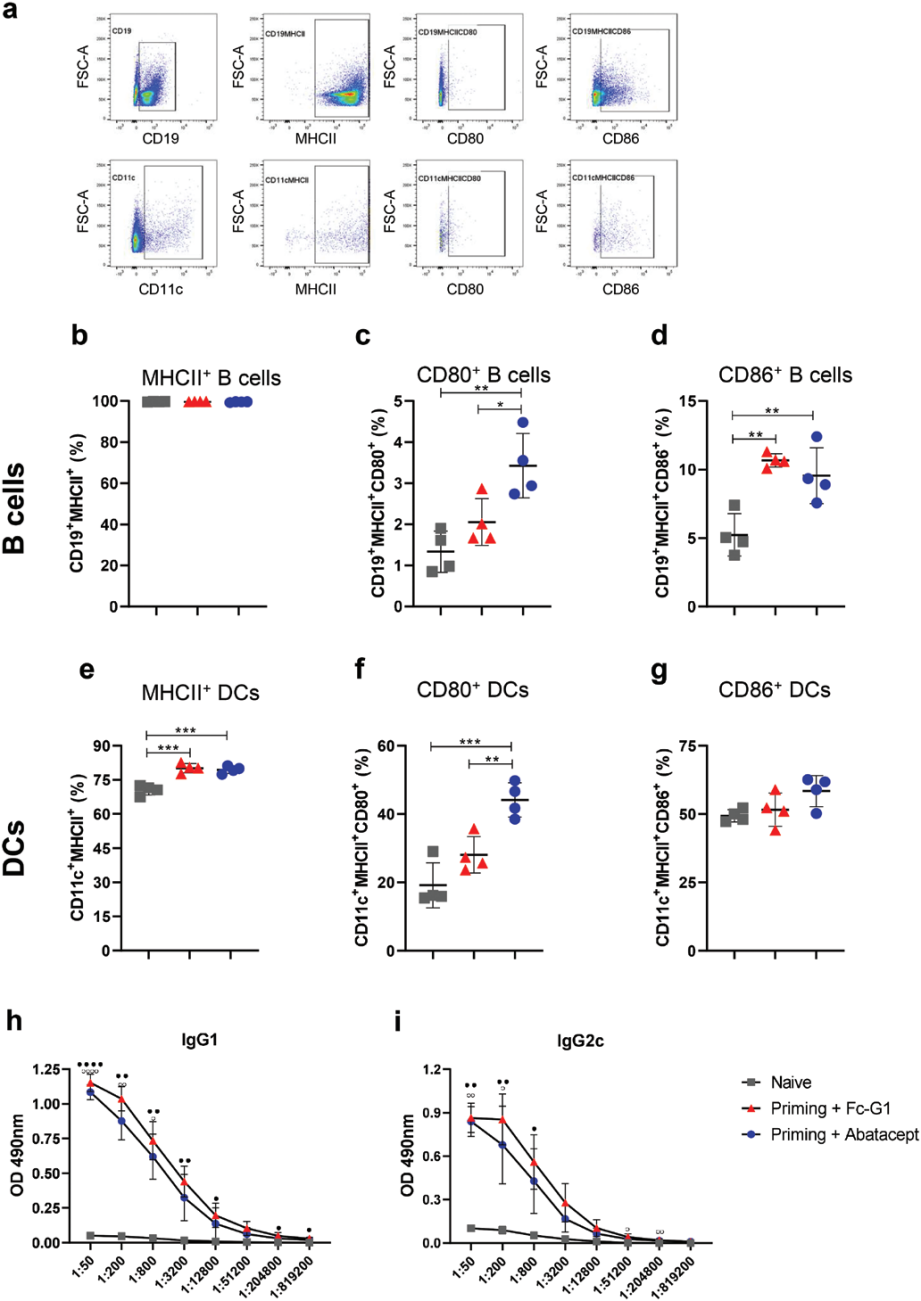
**Effect of abatacept on APC subsets and anti-OVA IgG levels during established immune responses.** Following the protocol described previously in Figure 1F, cells from popliteal lymph nodes were stained for B cell and DC markers to be detected by flow cytometry. (**A**) Single, live CD19^+^ (B Cells – top) and CD11c^+^ (DCs – bottom) were distinguished and analysed for their expression of MHCII^+^; CD80^+^ and CD86^+^. (**B**) Percentage of B cells expressing MHCII^+^. (**C**) Percentage of CD19^+^MHCII^+^ cells expressing CD80^+^. (**D**) Percentage of CD19^+^MHCII^+^ cells expressing CD86^+^. (**E**) Percentage of DCs expressing MHCII^+^. (**F**) Percentage of CD11c^+^MHCII^+^ cells expressing CD80^+^. (**G**) Percentage of CD11c^+^MHCII^+^ cells expressing CD86^+^. *P < 0.05; **P < 0.01; ***P < 0.001; ****P < 0.0001. (**H**) Anti-OVA IgG1 levels in serum collected 7 days after HAO challenge. (**I**) Anti-OVA IgG2c levels in serum collected 7 days after HAO challenge. *P < 0.05; **P < 0.01; ***P < 0.001; ****P < 0.0001 – for Fc-G1 in comparison to Naïve. °*P* < 0.05; °°*P* < 0.01; °°°*P* < 0.001; °°°°*P* < 0.0001 – for Abatacept in comparison to Naïve. Grey squares represent naïve mice; red triangles represent mice injected with Fc-G1, and blue circles represent mice injected with abatacept. Data were analysed by one- or two-way ANOVA, followed by post hoc Tukey’s test, for multiple comparisons; *n* = 4, over 1 experiment.

One of the effects previously described for abatacept is the reduction in the conditioning of DCs [8]. Thus, the phenotypes of APCs were also analysed: the subpopulations examined were CD19^+^ cells (B cells) and CD11c^+^ cells (DCs), and both these populations were assessed for the expression of MHC class II. As abatacept is a CTLA-4Ig molecule, its function is exerted by binding to CD80 and CD86 molecules. Therefore, we also analysed the expression of CD80 and CD86 on CD19^+^MHCII^+^ and CD11c^+^MHCII^+^ cells (Fig. 2a).

Almost 100% of CD19^+^ cells expressed MHCII, and there was no difference between the groups (Fig. 2b). Interestingly, treatment with abatacept caused a significant increase in the percentage of B cells expressing CD80^+^ (Fig. 2c) when compared with mice that received Fc-G1. The percentage of CD86^+^ B cells, on the other hand, was significantly increased in both abatacept- and Fc-G1-treated mice, in comparison to naïve mice (Fig. 2d). As for DCs, the percentages of CD11c^+^MHCII^+^ cells (Fig. 2e) were significantly increased in mice that were immunized, compared with naïve mice, and there was no difference between treated groups. As observed for B cells, there was a significant increase in the percentage of DCs expressing CD80^+^ in mice that received abatacept, compared with the other groups (Fig. 2f). However, no differences were observed in the frequency of DCs expressing CD86^+^ (Fig. 2g).

Another effect of abatacept treatment is to prevent CD4^+^ T cells from becoming competent T_fh_ cells, impairing T-/B-cell communication, and consequently reducing antibody production [7]. Thus, to investigate whether this effect was also observed for abatacept administration during an established immune response, serum was collected from mice 7 days after HAO injection and levels of anti-OVA IgG1 and IgG2c were measured by ELISA. Comparison between the groups showed higher levels of anti-OVA IgG1 (Fig. 2h) and IgG2c (Fig. 2i) in the groups that received OVA/CFA, followed by HAO injections, compared with naïve mice. However, there was no difference between treatments (abatacept and control Fc-G1).

### The effect of abatacept on CD4 T-cell activation profile is not long-lived *in vivo*

After confirming abatacept impaired T-cell activation during priming and during established immune responses, we investigated the duration of these effects.

**Figure 3:**
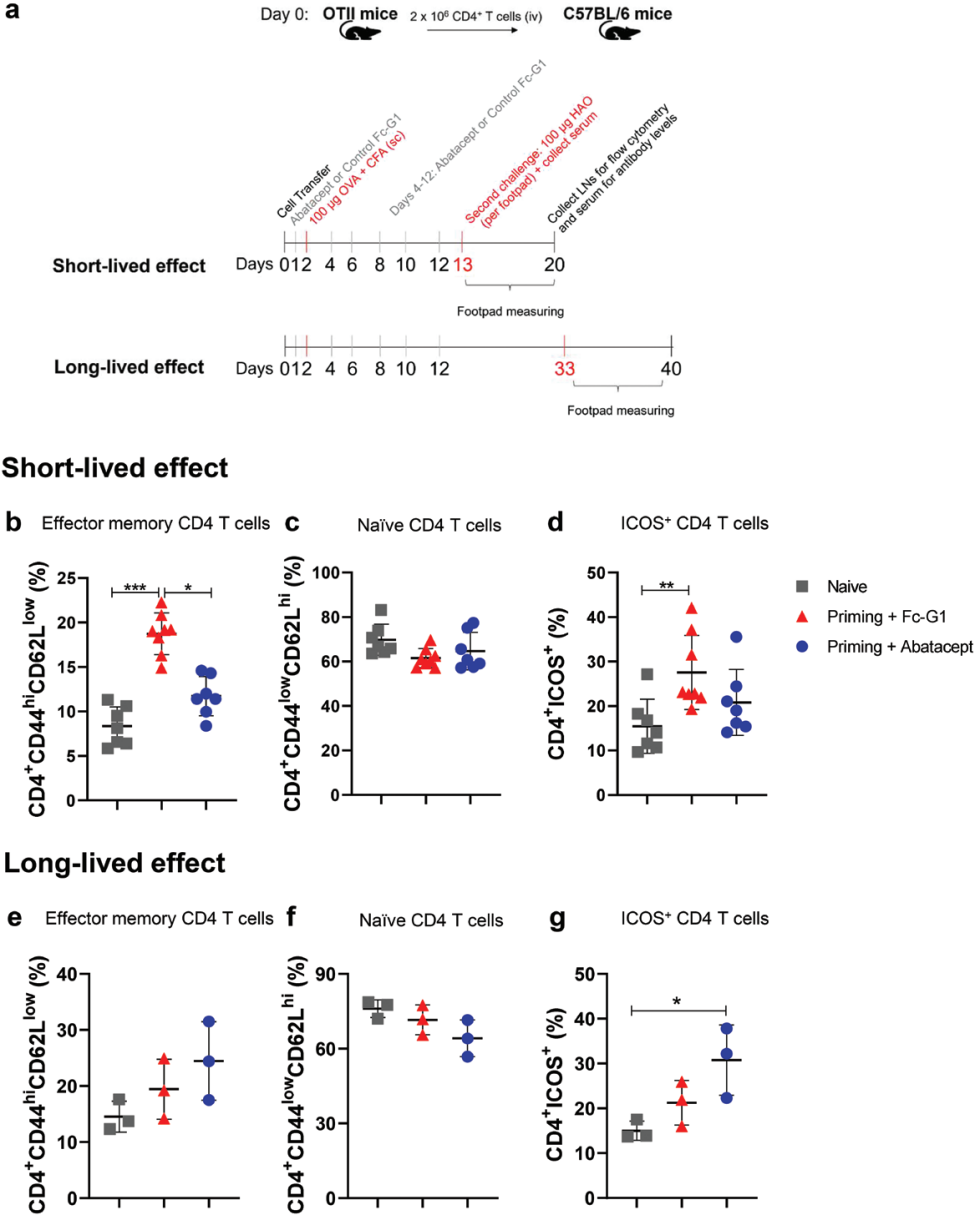
**The effect of abatacept on CD4 T cell activation profile is not long-lived *in vivo*. (A**) OTII cells were transferred into female C57BL/6 mice. After one day, mice were injected with either abatacept or Fc-G1 and, 24 h later, immunized with OVA/CFA. For the posterior 10 days, mice received abatacept or Fc-G1 every other day. To investigate the short- and long-lived effect of abatacept, mice were challenged with HAO into the footpads either 24 h or 21 days after the last injection, respectively. Negative control mice (Naïve group) were not immunized or challenged. Seven days after the challenge, popliteal lymph nodes were collected for flow cytometry and blood serum for the detection of anti-OVA IgG levels. Cells from mice (*n* = 7–8, over two experiments) challenged 24 h after the last abatacept/Fc-G1 injection were analysed for the percentage of (**B**) Effector memory CD4^+^ T cells. (**C**) Naïve CD4^+^ T cells. (**D**) CD4^+^ T cells expressing ICOS. Cells from mice (*n* = 3, over one experiment) challenged 21 days after the last abatacept/Fc-G1 injection were analysed for the percentage of (**E**) Effector memory CD4^+^ T cells. (**F**) Naïve CD4^+^ T cells. (**G**) CD4^+^ T cells expressing ICOS. Grey squares represent naïve mice; red triangles represent mice injected with Fc-G1, and blue circles represent mice injected with abatacept. Data were analysed by one-way ANOVA, followed by post hoc Tukey’s test, for multiple comparisons. *P < 0.05; **P < 0.01; ***P < 0.001; ****P < 0.0001.

OTII cells were transferred into C57BL/6 mice, which were then treated with abatacept or control Fc-G1 (via ip). One day later, they were immunized with OVA/CFA in the scruff of the neck, followed by abatacept or Fc-G1 injections every 2 days for 10 days. To examine the duration of the effect of abatacept treatment, two timelines were designed after the last injection of abatacept or Fc-G1. For the first, the HAO challenges were administered into the footpads one day later (‘short-lived’), and for the second, HAO injections were administered 21 days after the last injection of abatacept or Fc-G1 (‘long-lived’). For 3 days post HAO injections, footpad thickness was measured, and pLNs were collected for flow cytometry 7 days post HAO challenge (Fig. 3a). Naive mice, which were not immunized and did not receive any treatment, were used as negative controls.

As observed previously [8], when there was no interval between abatacept or Fc-G1 administration and HAO challenge (short-lived protocol), the percentage of effector memory CD4^+^ T cells from the draining lymph nodes of mice treated with abatacept was significantly reduced when compared with Fc-G1-injected mice (Fig. 3b), while the percentages of naïve CD4^+^ T cells were similar between the groups (Fig. 3c). Moreover, the percentage of CD4^+^ T cells expressing ICOS was significantly higher in mice injected with Fc-G1, compared with naïve mice. The percentage of ICOS^+^ CD4^+^ T cells was decreased in abatacept-treated mice (ns; Fig. 3d).

However, when a 21-day interval was added between the last day of treatment and HAO challenge (long-lived protocol), this protective effect was reversed. Mice treated with abatacept had an increased percentage of effector memory cells (ns; Fig. 3e) and proportionally decreased frequency of naïve CD4^+^ T cells (ns; Fig. 3f). The effect of reducing the percentage of ICOS^+^ CD4^+^ T cells was also lost (Fig. 3g).

For CD4^+^CD45.1^+^ cells, the retention of OVA-specific T cells after the treatment with abatacept was observed for both protocols (Supplementary Fig. S2a). Equally, there were also no differences between Fc-G1 or abatacept treatment on CD45.1^+^ cell phenotypes, for both protocols (Supplementary Fig. S2b).

In summary, these data indicate that the effect of abatacept treatment on total CD4^+^ T cells is short lived, as it is lost if the interval between treatment and challenge is increased.

### Initial abatacept treatment is associated with reduced DC conditioning, but this effect is not long-lived *in vivo*

**Figure 4:**
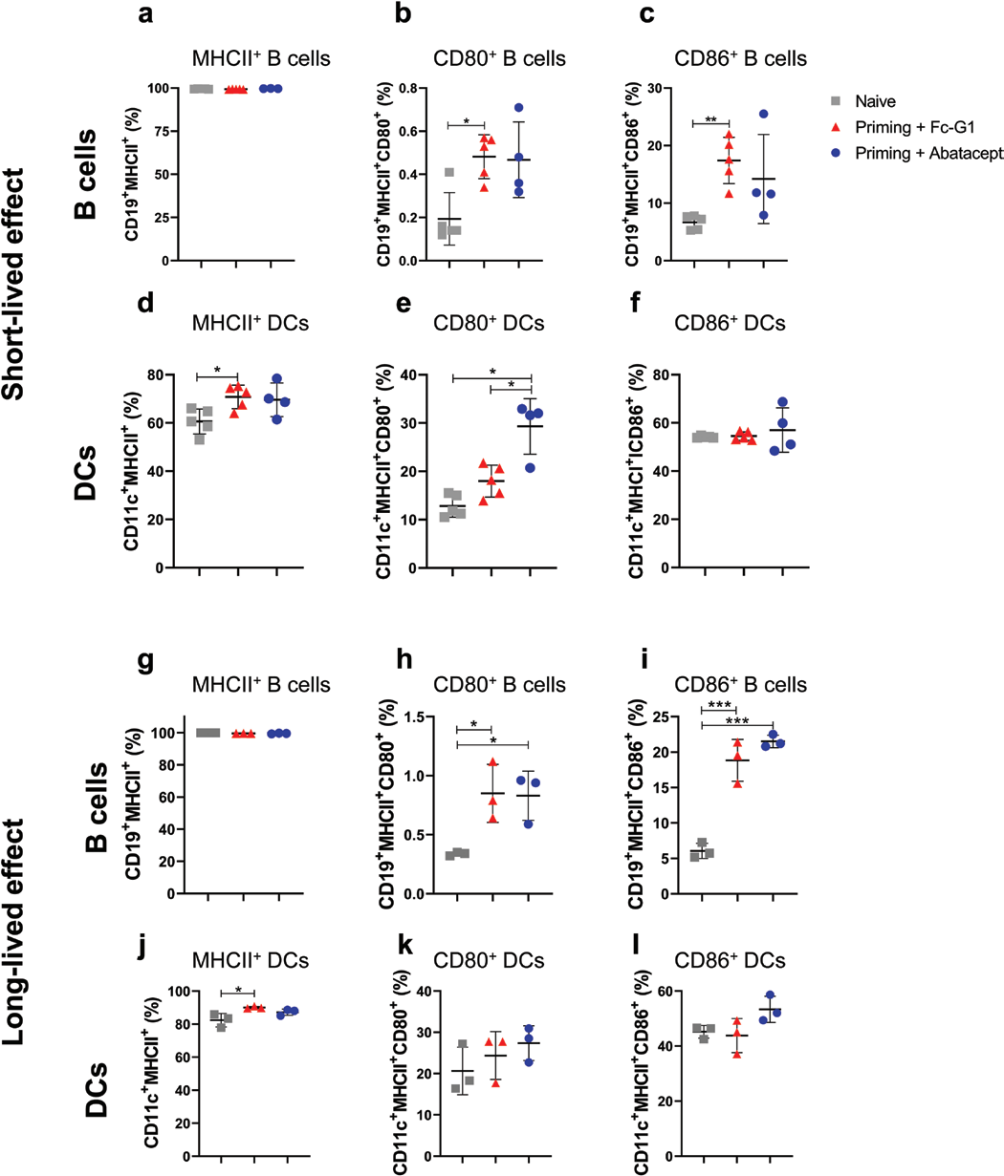
**Initial Abatacept treatment is associated with reduced DC conditioning, but this effect is not long-lived *in vivo*.** Following the protocol described previously in Figure 3A, cells from popliteal lymph nodes were stained for B cell and DC markers to be detected by flow cytometry. Cells from mice (*n* = 4–5, over one experiment) challenged 24 h after the last abatacept/Fc-G1 injection were analysed for (**A**) Percentage of B cells expressing MHCII^+^. (**B**) Percentage of CD19^+^MHCII^+^ cells expressing CD80^+^. (**C**) Percentage of CD19^+^MHCII^+^ cells expressing CD86^+^. (**D**) Percentage of DCs expressing MHCII^+^. (**E**) Percentage of CD11c^+^MHCII^+^ cells expressing CD80^+^. (**F**) Percentage of CD11c^+^MHCII^+^ cells expressing CD86^+^. Cells from mice (*n* = 3, over one experiment) challenged 21 days after the last abatacept/Fc-G1 injection were analysed for (**G**) Percentage of B cells expressing MHCII^+^. (**H**) Percentage of CD19^+^MHCII^+^ cells expressing CD80^+^. (**I**) Percentage of CD19^+^MHCII^+^ cells expressing CD86^+^. (**J**) Percentage of DCs expressing MHCII^+^. (**K**) Percentage of CD11c^+^MHCII^+^ cells expressing CD80^+^. (**L**) Percentage of CD11c^+^MHCII^+^ cells expressing CD86^+^. Grey squares represent naïve mice; red triangles represent mice injected with Fc-G1, and blue circles represent mice injected with abatacept. Data were analysed by one-way ANOVA, followed by post hoc Tukey’s test, for multiple comparisons. *P < 0.05; **P < 0.01; ***P < 0.001; ****P < 0.0001.

We next determined the effects of both experimental protocols (‘short-lived’ and ‘long-lived’ protocols) on the phenotype of APCs. For the short-lived effect experiment, almost all B cells expressed MHCII and there was no difference between the groups (Fig. 4a). However, the HAO challenge led to increased frequency of the costimulatory molecules CD80 (Fig. 4b) and CD86 (Fig. 4c) in both groups compared with naïve mice, not being dependent on treatment. For DCs, there was a significant increase in the percentage of CD11c^+^ cells expressing MHCII^+^ (Fig. 4d), when compared with naïve mice. Interestingly, the percentage of DCs expressing CD80 increased in mice treated with abatacept, in comparison to naïve and Fc-G1-treated mice (Fig. 4e), while the percentages of DCs expressing CD86 were similar between the groups (Fig. 4f).

When the HAO challenge was 21 days after the cessation of treatment with abatacept or Fc-G1 (long-lived protocol), B-cell expression of MHCII^+^ was similar to those observed for the ‘short-lived’ protocol with percentages close to 100% in all three groups (Fig. 4g). However, while both treated groups showed increased percentage of B cells expressing CD80^+^ (Fig. 4h) and CD86^+^ (Fig. 4i), in comparison to naïve mice, there were no differences between abatacept and Fc-G1 treatments. As observed in the short-lived protocol, in mice treated with control Fc-G1, there was an increased frequency of DCs expressing MHCII^+^, compared with naïve mice in the 21-day interval timeline (Fig. 4j). The percentage of DCs expressing CD80^+^ was similar between the groups (Fig. 4k) and the increase observed in mice treated with abatacept in the short-lived protocol was lost in the long-lived protocol. The percentage of DCs expressing CD86^+^ was higher in mice treated with abatacept, but this was not statistically significant (ns; Fig. 4l). These results suggest that, like the abatacept effect on CD4^+^ T cells (Fig. 3), the effect on both B cells and DCs is also short lived.

### Abatacept treatment reduced antibody responses to OVA immunization but only when the HAO challenge was 24 h after the treatment

**Figure 5:**
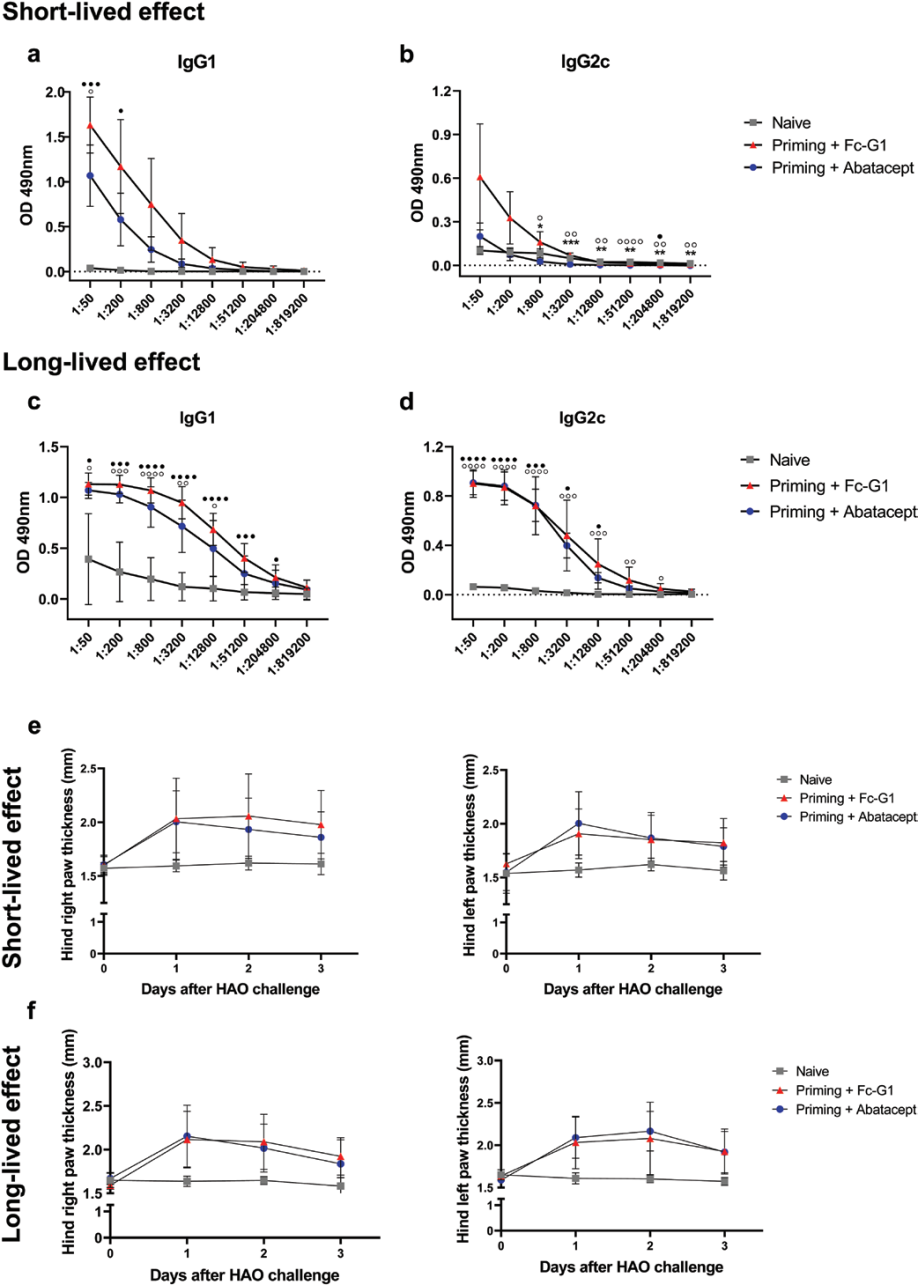
**Short- and Long-lived effect of abatacept on anti-OVA IgG levels and clinical signs of inflammation.** Following the protocol previously described in Figure 3A, serum from mice (*n* = 4–5, over one experiment) challenged 24 h after the last abatacept/Fc-G1 injection were analysed for (**A**) anti-OVA IgG1 levels and (**B**) anti-OVA IgG2c levels. Serum from mice (*n* = 7, over two experiments) challenged 21 days after the last abatacept/Fc-G1 injection were analysed for (**C**) anti-OVA IgG1 levels and (**D**) anti-OVA IgG2c levels. *P < 0.05; **P < 0.01; ***P < 0.001; ****P < 0.0001 – Fc-G1 in comparison to Naïve. °*P* < 0.05; °°*P* < 0.01; °°°*P* < 0.001; °°°°*P* < 0.0001 – Abatacept in comparison to Naïve. • *P* < 0.05; •• *P* < 0.01; ••• *P* < 0.001; •••• *P* < 0.0001 – Abatacept in comparison to IgG1. Right and left, respectively, hind paw thickness was measured for 3 days after HAO injection in mice challenged (**E**) 24 h after the last abatacept/Fc-G1 injection and (**F**) 21 days after the last abatacept/Fc-G1 injection (*n* = 7–8, over two experiments). Grey squares represent naïve mice; red triangles represent mice injected with Fc-G1, and blue circles represent mice injected with abatacept. Data were analysed by one- or two-way ANOVA, followed by post hoc Tukey’s test, for multiple comparisons.

When the HAO challenge was one day after the last abatacept or Fc-G1 injection (short-lived protocol), the levels of IgG1 (Fig. 5a) and IgG2c (Fig. 5b) were higher in all immunized mice, compared with naïve mice. However, the levels were significantly lower for mice that were treated with abatacept, compared with Fc-G1. On the other hand, when the HAO challenge happened 21 days after the last abatacept or Fc-G1 treatment, the levels of IgG1 (Fig. 5c) and IgG2c (Fig.5d) anti-OVA antibodies were significantly higher than in naïve mice and there was no difference between treatment groups.

For 3 days after the HAO challenge, footpads were measured with a calliper for the assessment of swelling. The challenge provoked increased footpad thickness in all immunized groups, compared with naïve mice in both protocols. However, there was no significant difference between these groups for either protocol (Fig. 5e and f).

### *In vivo* persistence of antigen leads to loss of abatacept function after treatment cessation

**Figure 6:**
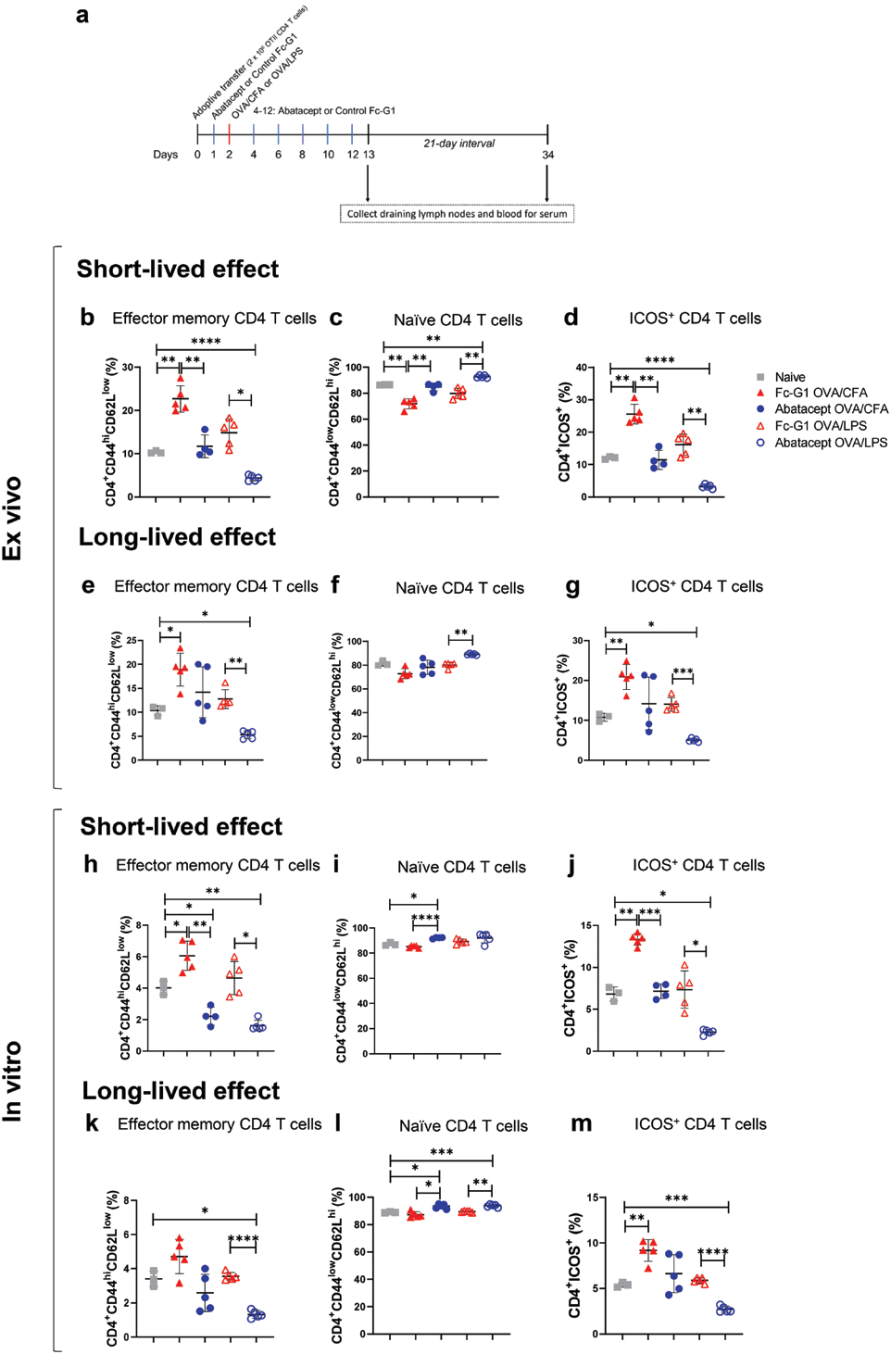
**Constant presence of antigen leads to loss of abatacept function after treatment is ceased.** (**A**) OTII cells were transferred into female C57BL/6 mice. After one day, mice were injected with either abatacept or Fc-G1 and, 24 h later, immunized with OVA/CFA or OVA/LPS. For the posterior 10 days, mice received abatacept or Fc-G1 every other day. To investigate the short- and long-lived effect of abatacept, blood and draining lymph nodes were collected either 24h or 21 days after the last injection, respectively. Negative control mice (Naïve group) were not immunized. For the *ex vivo* short-lived timeline: (**B**) Percentage of effector memory CD4^+^ T cells. (**C**) Percentage of naïve CD4^+^ T cells. (**D**) Percentage of CD4^+^ T cells expressing ICOS. For the *ex vivo* long-lived timeline: (**E**) Percentage of effector memory CD4^+^ T cells. (**F**) Percentage of naïve CD4^+^ T cells. (**G**) Percentage of CD4^+^ T cells expressing ICOS. For *in vitro* experiments, draining lymph node cells were restimulated with OVA peptide for 48 h. Short-lived timeline: (**H**) Percentage of effector memory CD4^+^ T cells. (I) Percentage of naïve CD4^+^ T cells. (**J**) Percentage of CD4^+^ T cells expressing ICOS. Long-lived timeline: (**L**) Percentage of effector memory CD4^+^ T cells. (**L**) Percentage of naïve CD4^+^ T cells. (**M**) Percentage of CD4^+^ T cells expressing ICOS. *n* =3–5, done over one experiment. Grey squares represent naïve mice; red-filled triangles represent mice injected with Fc-G1 and immunized with OVA/CFA; blue-filled circles represent mice injected with abatacept and immunized with OVA/CFA; red empty triangles represent mice injected with Fc-G1 and immunized with OVA/LPS and blue empty circles represent mice injected with abatacept and immunized with OVA/LPS. Data were analysed by one-way ANOVA, followed by post hoc Tukey’s test, for multiple comparisons. *P < 0.05; **P < 0.01; ***P < 0.001; ****P < 0.0001.

With the observation that the abatacept effect is lost after treatment is ceased, we aimed to characterize the activation state of CD4^+^ T cells at the time mice would be challenged in the footpad and understand how the long-term persistence (OVA emulsified in CFA) versus a brief presence (OVA in combination with LPS) [15] of antigen available *in vivo* for presentation to CD4^+^ T cells affect the efficacy of abatacept. For this purpose, OTII cells were transferred into naïve C57BL/6 mice. The next day, mice were treated with either abatacept or Fc-G1 and one day later, mice were immunized with either OVA/CFA or OVA/LPS. Every 2 days after immunization, mice were treated with abatacept and Fc-G1 until day 12. Blood and draining lymph nodes (axillary, brachial, and inguinal) were collected on day 13 for the short-lived effect and on day 34 (21 days after the last abatacept treatment) for the long-lived effect (Fig. 6a).

When CD4^+^ T cells were analysed one day after the last injection of abatacept, the percentage of CD4^+^CD44^hi^CD62L^low^ effect memory cells was reduced in mice treated with abatacept, compared with Fc-G1, for both the OVA/CFA and OVA/LPS immunized mice (Fig. 6b). Proportionally, the percentage of naïve CD4^+^ T (CD4^+^CD44^low^CD62L^hi^) cells were increased in both these groups (Fig. 6c). There was also a reduction in the percentage of CD4^+^ T cells expressing ICOS for both immunized groups treated with abatacept (Fig. 6d), supporting previous observations of the effect of abatacept.

By contrast, for mice in which the abatacept treatment ceased 21 days before the end of the experiment (long-lived protocol), the percentage of effector memory CD4^+^ T cells was only significantly reduced in comparison to Fc-G1 for mice immunized with OVA/LPS (Fig. 6e). Similarly, the percentage of naïve cells were increased and ICOS expression significantly decreased only in abatacept mice treated immunized with OVA/LPS (Fig. 6f and g, respectively).

Analysing OVA-specific CD4^+^ T cells, the percentages of CD4^+^CD45.1^+^ cells were higher in mice treated with abatacept in both protocols (Supplementary Fig. S3a), and the variations in the phenotypes were similar to those observed in total CD4^+^ T cells (Supplementary Fig. S3b).

We also evaluated these CD4^+^ T cells after restimulation *in vitro*. At the end of both protocols, draining lymph node cells were cultured with OVA peptide for 48 h and evaluated by flow cytometry. The results from *ex vivo* cells were confirmed: for the short-lived protocol, the percentage of effector memory T cells decreased in immunized mice treated with abatacept, compared with mice treated with Fc-G1 and naïve mice (Fig. 6h). For mice treated with abatacept and immunized with OVA/CFA, there was also an increased percentage of naïve cells (Fig. 6i). As expected, the percentages of CD4^+^ T cells expressing ICOS also decreased in mice treated with abatacept, for both modes of immunization (Fig. 6j). When the effect of abatacept was evaluated 21 days after the last treatment, the statistically significant differences between abatacept- and Fc-G1-treated in mice immunized with OVA/CFA were lost for the percentage of effector CD4^+^ T cells (Fig. 6k) and T cells expressing ICOS (Fig. 6l). However, there was still a significant reduction for both these populations when mice were immunized with OVA/LPS. For the *in vitro* assay, the percentage of CD4^+^ naïve T cells was significantly higher in mice treated with abatacept for both immunization types (Fig. 6m).

Anti-OVA IgG1 and IgG2c levels were also measured, but the values were below the detection limit for the short-lived effect groups (data not shown) and the comparison between short- and long-lived timelines was therefore not possible.

## Discussion

Abatacept, a CTLA-4Ig costimulatory blockade that interferes with CD28 on T cells binding to CD80/CD86 on APCs, has shown efficacy in clinical trials for RA [16] (which has become one of the established treatments), psoriatic arthritis [17], and juvenile idiopathic arthritis [18], among other autoimmune diseases.

The mechanisms of action of abatacept have been studied by our group previously, demonstrating that blocking the interaction of CD28 with CD80/CD86 retains the CD4 T cells in a state between TCR engagement and priming, denominated T_induced naïve_, which also impairs DC conditioning [8]. Abatacept has also a major effect on T follicular helper (Tfh) cells, disabling their migration and communication with B cells in the B-cell area of lymph nodes and consequently inhibiting antibody production [7]. The impact on Tfh cells was also studied in a model of type 1 diabetes, showing that T_fh_ and other ICOS^+^ CD4^+^ T cell subsets are the most sensitive to the effect of abatacept [19]. Our current project has supported these features, showing a reduced percentage of activated CD4 T cells and a lower percentage of cells expressing ICOS, after a single injection of abatacept.

We also observed similar results when abatacept was administered during an established immune response. However, in this case, the effect on CD4 T cells was not followed by reduced anti-OVA IgG levels in treated mice. The lack of modulation of all the parameters studied may indicate that timing of administration is essential for the full potential of this drug. Clinical trials focussing on treatment for undifferentiated inflammatory arthritis and ‘very early’ RA (less than 2 years of RA symptoms), for example, showed better results that were sustained for longer periods of time [20].

One interesting finding in this study was the different effects of abatacept on CD80^+^ and CD86^+^ APCs. It is important to note that this was an unexpected finding, as other studies have shown reduced frequency of CD80^+^-expressing APCs after abatacept treatment. The reduction could be explained by either the binding of abatacept to CD80 and CD86 hindering flow cytometry antibodies and/or internalization by APCs [21]. Considering the findings by Kennedy *et al*. [22], one could hypothesize that as the binding of CD86 to abatacept is more affected by pH changes, resulting in de-coupling, CD86 would then be more susceptible to membrane-bound-CTLA-4 transendocytosis, while CD80 would be ‘protected’ while bound to abatacept. Another form of protection for CD80-expressing cells would be the *cis*-heterodimerization of these molecules to PD-L1 on the surface of APCs, also avoiding transendocytosis [23]. Further studies would be necessary to determine the existence of the said heterodimerization on the surface of these DCs and B cells.

The main aim of our study, however, was to investigate the duration of the effect of abatacept. While it has proven efficacy during treatment, less is known about how long this effect is maintained after the administration ceases. Although drug-free remission is observed in some patients—and the presence of abatacept in the treatment regime increases the chance of remission—the numbers are low and decrease with time [20, 24].

Our results demonstrate that while short-lived immunomodulation was observed (reduced percentage of effector and ICOS^+^ CD4^+^ T cells and lower levels of anti-OVA IgG), when the HAO challenge happened 21 days after the last abatacept treatment, this protective effect was lost. As with most autoimmune diseases, the obstacle to achieving drug-free remission in RA is that treating an ongoing process does not alter the initial breach of self-tolerance or the subsequent cascade onwards [25]. For abatacept, this could mean that while the costimulatory blockade affects CD4^+^ T cells during treatment, when administration ceases, it will not prevent a new wave of priming for T cells with a different specificity. Not only that but having such an important effect on T_fh_, abatacept administration interruption may reinstate antibody production (as observed with our long-lived timeline).

To investigate the influence of a constant versus a transient form of antigen presentation and characterize the CD4^+^ T cells pre-challenge, we analysed the effect of abatacept after the immunization with OVA either emulsified in CFA or combined with LPS in the short- and the long-lived timelines. Freund’s adjuvant is known to sustain continuous antigen presentation for several weeks, supporting a potent immune response [26]. In this sense, it resembles what happens in autoimmune diseases where the release of autoantigens is continuous. Although abatacept reduced the frequency of effector and ICOS^+^ CD4^+^ T cells *in vivo* and *in vitro* for both OVA/CFA and OVA/LPS immunizations when the CD4^+^ T cells were analysed 24 h after the last abatacept treatment, these reductions were only observed in abatacept-treated mice immunized with OVA/LPS 21 days after the last injection.

In conclusion, abatacept impairs T-cell priming and consequently affects antibody production by inhibiting mostly ICOS^+^ T cells. This protection, however, is short lived in the presence of antigen and lost once the drug is withdrawn, supporting the requirement for long-term administration in patients. It is known that new-generation CTLA-4-Ig has been designed to achieve higher affinity with CD80 and CD86, as well as a less-frequent regimen of administration [27], which could mean that their long-lived effect is also improved. The combination of abatacept with other immunomodulators has also been tested in animal models and clinical trials (the latter with precautions to side effects). In a model of type 1 diabetes, abatacept treatment combined with IL-2 administration had the beneficial effect of restoring regulatory T-cell homeostasis [10]. Although the combination of Janus kinase (Jak) inhibitors and abatacept is not usually recommended, there are already case observations of difficult-to-treat patients with RA where they were more effective together than as a monotherapy [28]. Future studies will be important to elucidate whether the combination of abatacept with other drugs could not only improve its efficacy but also the effect duration. Considering our hypothesis that the constant presence of new autoantigens is what prevents the effect of abatacept from being long lived, any treatment that can revert the breach of self-tolerance may be an optimal combination.

## Supplementary Material

kyad029_suppl_Supplementary_Figures_S1

kyad029_suppl_Supplementary_Figures_S2

kyad029_suppl_Supplementary_Figures_S3

## Data Availability

The data underlying this article will be shared upon reasonable request to the corresponding author.

## Abbreviations

CTLA-4: cytotoxic T-lymphocyte-associated protein 4
DCs: dendritic cells
DTH: delayed-type hypersensitivity
HAO: heat-aggregated OVA
Jak: Janus kinase
LPS: lipopolysaccharides
MHC: major histocompatibility complex
pLN: popliteal lymph nodes
sc: subcutaneously
TCR: T-cell receptor
T_fh_: T follicular helper cell
OVA: , ovalbumin
