# Supplementary figures and images for: The effect of abatacept on T-cell activation is not long-lived in vivo
Source: Discov Immunol. 2024 Jan 4;3(1):kyad029. doi: 10.1093/discim/kyad029 (PMC10917171; doi:10.1093/discim/kyad029)

S1

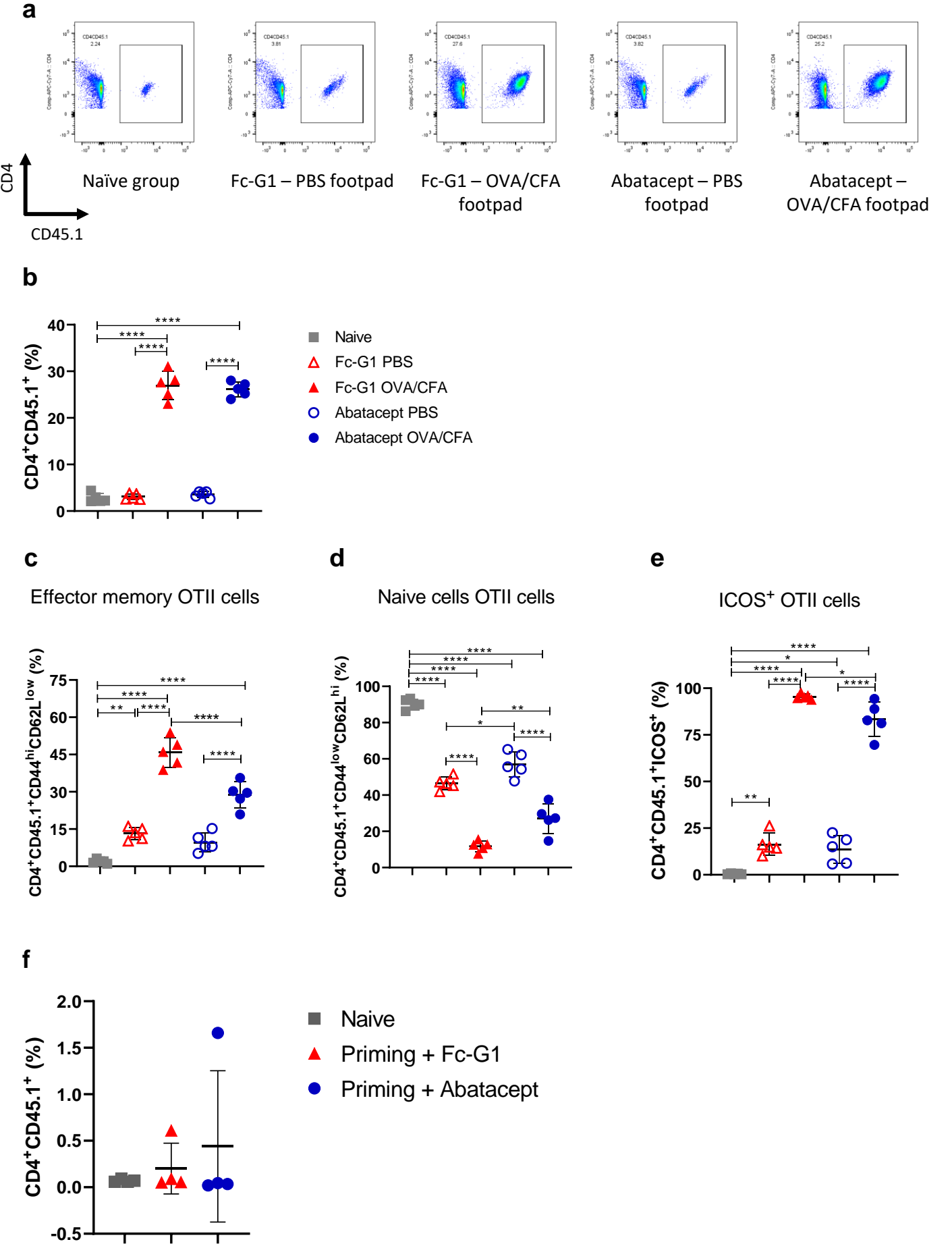

Supplement: kyad029_suppl_Supplementary_Figures_S1 [file kyad029_suppl_supplementary_figures_s1.pdf]

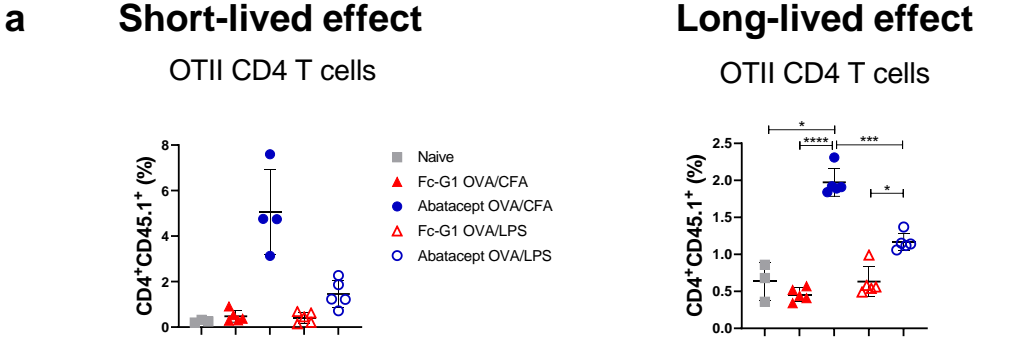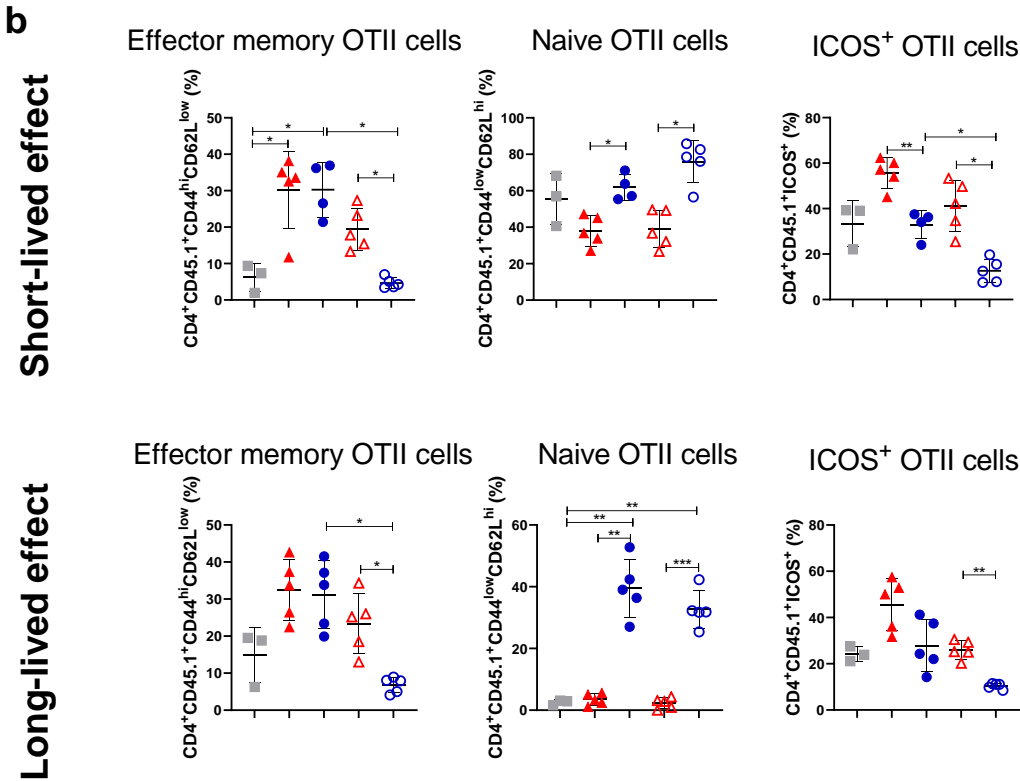

Supplement: kyad029_suppl_Supplementary_Figures_S3 [file kyad029_suppl_supplementary_figures_s3.pdf]
