# Supplementary material for: The effect of abatacept on T-cell activation is not long-lived in vivo
Source: Discov Immunol. 2024 Jan 4;3(1):kyad029. doi: 10.1093/discim/kyad029 (PMC10917171; doi:10.1093/discim/kyad029)
Supplement: kyad029_suppl_Supplementary_Figures_S2 [file kyad029_suppl_supplementary_figures_s2.pdf]

S2

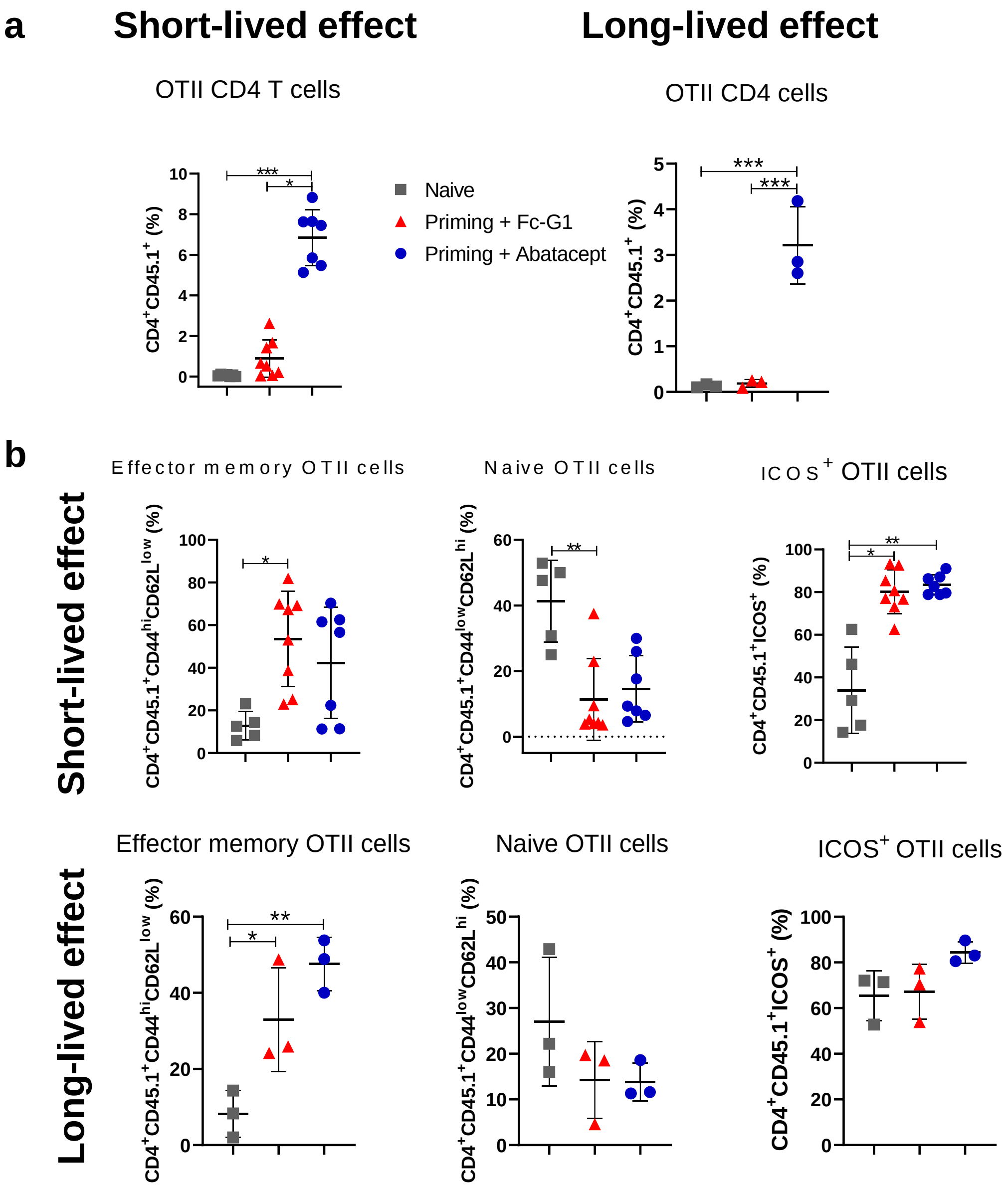

a
Short-lived effect
Long-lived effect
OTII CD4 T cells
OTII CD4 cells
CD4+CD45.1+ (%)
Naive
Priming + Fc-G1
Priming + Abatacept
***
*
b
Short-lived effect
Long-lived effect
Effector memory OTII cells
Naive OTII cells
ICOS+ OTII cells
CD4+CD45.1+CD44hiCD62Llow (%)
CD4+CD45.1+CD44lowCD62Lhi (%)
CD4+CD45.1+ICOS+ (%)
**
